# Selenium in Bodily Homeostasis: Hypothalamus, Hormones, and Highways of Communication

**DOI:** 10.3390/ijms232315445

**Published:** 2022-12-06

**Authors:** Pamela Toh, Jessica L. Nicholson, Alyssa M. Vetter, Marla J. Berry, Daniel J. Torres

**Affiliations:** 1 Pacific Biosciences Research Center, School of Ocean and Earth Science and Technology, University of Hawaii at Manoa, Honolulu, HI 96822, USA; 2 Department of Cell and Molecular Biology, John A. Burns School of Medicine, University of Hawaii at Manoa, Honolulu, HI 96813, USA; 3 School of Human Nutrition, McGill University, Montreal, QC H3A 0G4, Canada

**Keywords:** homeostasis, hypothalamus, energy metabolism, neuroendocrine, selenium, selenoprotein

## Abstract

The ability of the body to maintain homeostasis requires constant communication between the brain and peripheral tissues. Different organs produce signals, often in the form of hormones, which are detected by the hypothalamus. In response, the hypothalamus alters its regulation of bodily processes, which is achieved through its own pathways of hormonal communication. The generation and transmission of the molecules involved in these bi-directional axes can be affected by redox balance. The essential trace element selenium is known to influence numerous physiological processes, including energy homeostasis, through its various redox functions. Selenium must be obtained through the diet and is used to synthesize selenoproteins, a family of proteins with mainly antioxidant functions. Alterations in selenium status have been correlated with homeostatic disturbances in humans and studies with animal models of selenoprotein dysfunction indicate a strong influence on energy balance. The relationship between selenium and energy metabolism is complicated, however, as selenium has been shown to participate in multiple levels of homeostatic communication. This review discusses the role of selenium in the various pathways of communication between the body and the brain that are essential for maintaining homeostasis.

## 1. Introduction

The hypothalamus is the central coordinator of homeostatic processes in the body, such as blood pressure regulation [1], temperature control [2], circadian rhythm [3], and energy balance [4]. Various organs and tissues throughout the body constantly communicate to the hypothalamus, oftentimes in the form of hormones, providing information on their status. In response, the hypothalamus sends signals back to the body through different ‘axes’ of communication and influences behavior through connections to other brain regions. Disruption of the mechanisms underlying these pathways can result in homeostatic disorder. A common example of this is the tendency of the hypothalamus to develop leptin resistance during obesity. Due to the inflammation and damage caused by the obese state, the hypothalamus becomes less responsive to the appetite suppressing effects of the adipose tissue-derived hormone leptin, and, thus, impeding the individual from losing weight [5]. Similar disruptions of hypothalamic function can have wide-ranging effects as the hypothalamus controls a multitude of physiological functions. As the obesity and diabetes pandemics continue to worsen [6,7], fully understanding the intricacies of energy homeostasis and the pathology of metabolic disorder is a top priority for researchers seeking to help improve human health.

Poor nutrition is undoubtedly a major contributor to the development of metabolic disorder [8,9]. While there is an extensive well of knowledge on the effects of macronutrients (e.g., carbohydrates, fats, and proteins), the importance of micronutrients (e.g., vitamins, minerals and other trace elements) and other related factors, such as nutrigenomics and gut microbiota dynamics, has become more apparent over the years [10,11,12,13,14,15,16,17]. The focus of the field has, accordingly, shifted towards pursuing precision nutrition as a basis for therapeutic intervention [18,19]. The essential trace element selenium is critical for health, especially brain function, and has garnered increasing attention in this regard [20,21,22]. Selenium is known to influence energy metabolism in various ways and abnormal selenium status has been tied to metabolic disorders [23,24,25,26]. Transgenic disruption of either selenium metabolism or direct targeting of selenoproteins has resulted in detrimental metabolic phenotypes in multiple studies [27,28,29,30,31,32,33]. Accumulating evidence has also begun to reveal an important supportive role for selenium within the hypothalamus [21,34,35]. This review discusses the axes of communication responsible for the hypothalamic maintenance of homeostasis, with a focus on energy metabolism, and summarizes the current knowledge of the involvement of selenium.

## 2. Overview of Selenium in Biological Function

The antioxidant trace element selenium maintains cellular physiology, largely by helping to prevent oxidative stress. It is used by cells to produce selenoproteins, a family of enzymes that participate in various redox reactions. To date, a total of 25 selenoproteins have been identified in mammals, although some variability exists between species. To synthesize selenoproteins, selenium must be incorporated into the 21st amino acid, selenocysteine (Sec), which is, essentially, a cysteine, wherein the sulfur atom is replaced by selenium. This elemental substitution results in a more efficient redox enzyme as, although cysteine is present in various antioxidant proteins as well, sec is more reactive, due to the chemical attributes of selenium. The valence electrons in a selenium atom are more tightly held than those of sulfur and selenium is, therefore, more prone to perform the nucleophilic attack involved in reductive reactions. Additionally, since selenium, as the heavier element, is much less capable of forming π bonds, the selenium–oxide species that forms after the reduction event is itself more readily reduced [36]. Although the high reactivity of selenium makes it a potent antioxidant, the drawback is that there is a higher risk of toxicity. Thus, selenium is handled in a tightly regulated manner throughout the body [37].

As an essential micronutrient, selenium must be obtained through the diet. It is typically found in fruits, vegetables, cereals, meats, eggs, dairy, and legumes [38,39]. For adult humans, the recommended daily allowance (RDA) of selenium is 55 µg/day, although the RDA is higher under certain conditions, such as during pregnancy [40]. After being ingested, selenium is digested and absorbed through the intestines, and, then, shuttled to the liver, the principal distributor of selenium [41]. In the liver, selenium can be packaged for transport to various organs, including to the kidneys for excretion. The primary mechanism through which selenium travels through the bloodstream to reach target tissues involves a unique selenoprotein, called selenoprotein P (SELENOP). Often referred to as the ‘selenium transporter’, SELENOP is distinct from other selenoproteins, in that it can contain up to ten Sec residues, rather than just one, and, in fact, has multiple functions, including heavy metal binding capabilities and possible enzymatic redox activity [42]. Circulating SELENOP is able to bind receptors, such as the low-density lipoprotein receptor-related protein 2 (LRP2), also known as megalin, in the kidneys, and LRP8, in the testes and brain, after which it is posited to become endocytosed. Cells can then metabolize SELENOP and recycle its Sec residues for *de novo* production of selenoproteins [43]. This mechanism allows for selenium to be safely distributed and delivered to areas of need.

**Figure 1:**
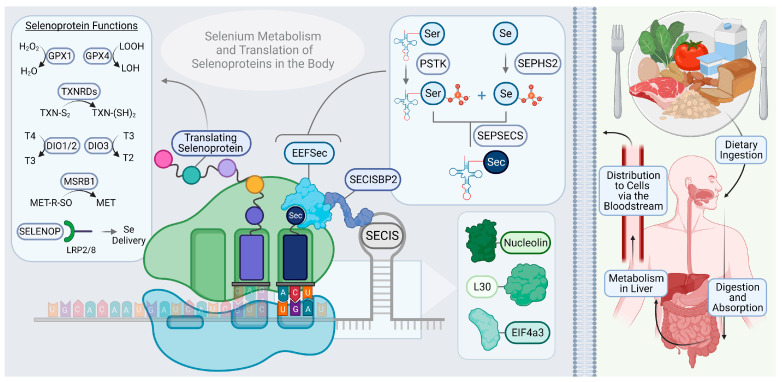
Diagram of dietary selenium metabolism and selenoprotein synthesis and function. Selenium is obtained through the diet and is commonly found in meats, eggs, dairy, grains, fruits, vegetables, nuts, and legumes. Following digestion and absorption through the gut, it is metabolized by the liver, and distributed throughout the body via the circulatory system. Inside the cell, selenophosphate synthetase 2 (SEPHS2) uses selenium (Se) to generate selenophosphate. PSTK (Protein serine threonine kinase) phosphorylates Ser-tRNASec, which is then converted into Sec-tRNASec by selenocysteine synthase (SEPSECS) using selenophosphate. The stem loop-containing selenocysteine insertion sequence (SECIS) in the selenoprotein mRNA is recognized by SECISBP2 (SECIS-binding protein 2), which helps stabilize the mRNA to allow for the recruitment of EEFSec (eukaryotic selenocysteine-specific elongation factor) and other members of the selenoprotein translation complex. The ribosomal protein L30, eukaryotic translation initiation factor a3 (eIFa3), and the nucleolus-resident phosphoprotein nucleolin have all been identified as SECIS-binding proteins, but their precise role in selenoprotein synthesis has not been well-elucidated [49]. The selenoproteins with the most well-characterized mechanisms of action include the glutathione peroxidases (GPXs), which reduce hydrogen peroxide (H_2_O_2_) and lipid hydroperoxides (LOOHs), the thioredoxin reductases (TXNRDs), the iodothyronine deiodinases (DIOs), which activate/de-activate thyroid hormones, methionine-R-sulfoxide reductase B1 (MSRB1), and the Se carrier selenoprotein P (SELENOP), which delivers selenium to cells through its interactions with LRP2 (low density lipoprotein receptor-related protein 2; also called megalin) and LRP8.

The unique process of selenoprotein synthesis relies on a cadre of dedicated molecular machinery [44]. In short, the incorporation of Sec requires the translational reprogramming of an UGA stop codon as Sec [45]. This requires the recognition of a stable loop structure within the selenoprotein, mRNA, called the Sec insertion sequence (SECIS), by the SECIS-binding protein 2 (SECISBP2). The recruitment of a complex of proteins follows, and includes, the UGA anticodon-containing Sec-tRNA^Sec^ (TRSP), which is needed to insert Sec [46]. The molecular mechanisms involved in selenoprotein synthesis are summarized in Figure 1. It is worth noting that there appears to exist a selenoprotein “hierarchy”, in which the synthesis of some selenoproteins, traditionally referred to as the “essential” selenoproteins, is prioritized over others when selenium supply is limited. This system is partially maintained by the affinity of SECISBP2 for the SECIS element, which can vary between selenoprotein mRNA species. For example, the affinity is much higher for the essential glutathione peroxidase 4 (GPX4) and thioredoxin reductase (TXNRD) sub-family but is lower for GPX1 [47]. Consequently, when TRSP levels are reduced, due to selenium scarcity, GPX4 and the TXNRDs are more capable of successfully recruiting TRSP and are, therefore, more preferentially translated. Selenoprotein mRNAs that lack Sec insertion at the UGA site are eventually be degraded via nonsense mediated decay [48]. These mechanisms have been adapted to effectively utilize selenium and maintain a favorable profile of selenoprotein expression within the cell. Through the production of these unique enzymes with covalently bound Sec, organisms can utilize the high reactivity of selenium to efficiently regulate redox balance.

Specific catalytic mechanisms of action have been confirmed for about half of the 25 known mammalian selenoproteins. These are the GPXs, the TXNRDs, methionine-R-sulfoxide reductase B1 (MSRB1), the iodothyronine deiodinases (DIOs), and selenophosphate synthetase 2 (SEPHS2), in addition to SELENOP, as described above. The first to be reported as a selenoprotein, GPX1, is one of the most ubiquitously expressed and is present in the cytoplasm and mitochondria, where it catalyzes the reduction of hydrogen peroxide [50,51,52]. This reaction involves the oxidation of the selenol in the selenocysteine residue of GPX1 by hydrogen, followed by its subsequent reduction by glutathione (GSH). Another member of the GPX sub-family, GPX4, is essential in many tissues as it primarily reduces lipid hydroperoxides [53]. In doing so, it helps prevent ferroptosis, a form of iron-dependent programmed cell death that has gained increasing attention since its discovery 10 years ago for its wide-ranging roles in physiology and its potential as a therapeutic target in human disease [54]. The TXNRDs catalyze the reduction of oxidized thioredoxin (TXN), a major disulfide reductase that is critical for cellular health, and are a generally highly expressed selenoprotein sub-family with strong therapeutic potential [55]. MSRB1 is responsible for reducing methionine sulfoxide (MetO) to methionine (Met). It supports a wide variety of cellular functions and recent reports elucidated a role for MSRB1 in the innate immune response [56]. The DIOs collectively regulate thyroid hormone activity [57]. DIO1 and DIO2 catalyze the conversion of the inactive thyroid hormone T4 to the active T3 form through the removal of an iodine atom. DIO3 carries out the deactivation of thyroid hormone by either reducing T4 into the inactive reverse T3 (rT3), or by converting T3 to T2 [58]. SEPHS2 is unique in that it directly partakes in selenium metabolism by helping to synthesize selenophosphate, which provides the selenium used to make Sec-loaded TRSP for selenoprotein translation [59].

In addition to the aforementioned selenoproteins, others have been determined to aid in various mechanisms and processes, but a specific enzymatic reaction has not been fully characterized and confirmed. For example, there are several selenoproteins localized to the ER that are involved in calcium (Ca^2+^) homeostasis and the endoplasmic reticulum (ER) stress response. These include DIO2, SELENOF, SELENOK, SELENOM, SELENON, SELENOS, and SELENOT [60]. SELENOF (previously known as SEP15) facilitates protein folding by mediating disulfide bond formation in glycoproteins, but has not yet been tied to Ca^2+^ homeostasis [61]. SELENOK plays a role in the ER associated protein degradation (ERAD) pathway and supports store-operated Ca^2+^ entry (SOCE) from the ER by facilitating the palmitoylation of inositol triphosphate receptors (IP3Rs) [62,63]. Although the mechanisms are unknown, SELENOM helps prevent excessive levels of cytosolic Ca^2+^ and appears to be a thiol-disulfide oxidoreductase [64]. SELENON senses ER luminal Ca^2+^ levels and, by acting as an oxidoreductase, activates the sarcoendoplasmic reticulum calcium transport ATPase (the SERCA pump) to replenish ER Ca^2+^ stores [65]. SELENOS participates in ERAD as a member of a multi-protein complex that facilitates the removal of misfolded peptides via retro-translocation followed by ubiquitin-mediated degradation [66]. Finally, SELENOT is thought to regulate Ca^2+^ homeostasis by acting on pumps and channels and may also be involved in ERAD [67,68]. These ER-resident selenoproteins may play an important role in preventing ER stress in the hypothalamus, which is thought to be a major contributor to metabolic disorder [69]. Table 1 summarizes the various selenoproteins and their known functions.

Selenium and selenoproteins support a wide variety of physiological functions in the body. Amongst the most well-described are its roles in fertility, thyroid gland physiology, and neurological function [70,71,72,73]. For example, selenium facilitates reproductive efficiency and GPX4 plays a crucial role in sperm motility [74]. There is also increasing evidence that it is important during pregnancy and for ovarian physiology [70]. The thyroid gland contains the highest level of selenium content per weight of all organs and is highly dependent on the actions of selenoproteins [75,76]. In addition to its ability to curb oxidative stress, selenium directly regulates thyroid hormone metabolism in the form of the DIO sub-family. Aside from these functions, selenium has been shown to support immunity, bone homeostasis, cardiovascular health, energy metabolism, and various others functions, and has also demonstrated anti-cancer, anti-inflammation, and anti-viral properties, including potentially protecting against SARS-CoV2 infection [77,78,79,80,81,82,83,84].

The brain relies heavily on selenium, due to its high metabolic rate and production of reactive oxygen species (ROS) [85]. Selenium is preferentially retained in the brain when dietary selenium is restricted, due to this high level of dependence [86,87]. Studies on animal models with disruptions to either selenoprotein expression or function have oftentimes yielded neurological phenotypes, typically involving neuromotor and memory deficits [88,89,90,91]. While the importance of selenium to neurodevelopment and neuronal health has been well-established, emerging evidence also implicates a critical role in adult neurogenesis, as well as the potential to directly influence neuronal activity by modulating redox signaling events [92,93,94]. In clinical studies, selenium deficiency has been implicated in multiple brain disorders in humans [95,96,97]. Selenium has been proposed as a supplemental therapy for the treatment of neurodegenerative diseases and strokes [98,99]. Recently, a prominent role for selenium in the hypothalamus has begun to come to light as well. This review discusses the influence of the essential trace element selenium, and the selenoproteins it is used to synthesize, in pathways of homeostatic communication. This topic is broken down into three main sections: (1) the physiology and inner workings of the hypothalamus, (2) the outward pathways from the hypothalamus that regulate homeostasis throughout the body, and (3) the routes of communication from the body to the hypothalamus.

## 3. Selenium in Hypothalamic Function

Interest in the role of selenium in hypothalamic health and function has grown in recent years. The hypothalamus is the central control system through which signals are sent to, and from, the brain to maintain physiological homeostasis. This vital brain structure is situated at the base of the brain and contains several anatomically and functionally distinct nuclei. The arcuate nucleus (ARC) lies at the most mediobasal aspect of the hypothalamus and is in direct contact with the median eminence (ME), an area with a loose and dynamic blood–brain barrier (BBB) [100]. The ME contains fenestrated capillaries that allow the diffusion of hormones and nutrients from the blood into the brain, where they can be detected by ‘first order neurons’ in the ARC [101]. The ARC is the most actively involved hypothalamic nucleus in directly receiving signals from the body and has been the focus of many recent investigations of selenium in the hypothalamus.

Two very prominent homeostatic sensory neuron populations that reside in the ARC are the agouti-related peptide (AGRP)-positive and pro-opiomelanocortin (POMC)-positive neurons [102]. These neurons express receptors for metabolic hormones, such as ghrelin, leptin, and insulin, and can also detect nutrients like glucose. In response, AGRP and POMC neurons adjust food intake and energy expenditure to help the organism meet the energy demands of its environment. AGRP neurons are particularly involved in these processes as their localization is concentrated nearest to the ME and there is evidence that many of them have projections that extend to the outside of the BBB making them extremely sensitive to fluctuations in the levels of circulating homeostatic signals [103,104]. The influence exerted by AGRP neuron activation, which promotes a positive energy balance, primarily by increasing feeding behavior, is in opposition to that of POMC neurons, which promote a negative energy balance by increasing energy expenditure. The activities of these two important neuronal populations often work synergistically, however, to regulate pituitary gland activity via the melanocortin system [105,106]. The hypothalamus, and particularly the ARC, is susceptible to oxidative stress, ER stress, and inflammation-mediated dysfunction under conditions of metabolic stress, such as the consumption of a high fat diet (HFD) [21,69,107,108,109]. It, therefore, stands to reason that protecting the hypothalamus would be a major mechanism through which selenium helps maintain energy balance.

One of the earliest reports on the role of selenium in the hypothalamus involved measurements of several trace elements in the rat hypothalamus during various states of reproductive function (e.g., proestrus and estrus of cycling females, castrated males, etc.). In this study, Merriam et al. observed that, while levels of iron, copper, zinc, arsenic, bromine, and rubidium all changed, only selenium levels stayed consistent across all conditions [110]. As noted by the authors, not much was known about the physiological functions of selenium at the time (the year 1979), which was preceded by the discovery of the first selenoprotein only several years prior [50,111]. Still, this finding was reminiscent of studies performed roughly a decade later showing that selenium is preferentially retained in the brain when dietary selenium is restricted [112]. Several other studies over the next couple of decades, following the report by Merriam et al., began unveiling the importance of selenium to pituitary gland function [113,114,115,116,117,118]. It was not until the 2000s, however, that a clearer picture of the role of selenium and selenoproteins in the hypothalamus itself began to develop.

Transcriptomic studies indicated that selenoproteins are abundantly expressed in the hypothalamus, especially AGRP and POMC neurons [119,120]. Selenoprotein gene expression in the hypothalamus was also noted to become altered by changes in diet and homeostatic signals. In the paraventricular nucleus (PVN), for example, 4 of the 100 genes most positively regulated in response to leptin are selenoproteins: *Gpx3*, *GPx4*, *Selenok*, and *Selenom* [121]. Although the physiological significance of these observations remains to be elucidated, their upregulation may serve to support increased neuronal activity levels, as leptin promotes the production and release of hypophysiotropic hormones from secretory neurons in the PVN that go on to act on the anterior pituitary. The increased *Gpx4* expression is noteworthy, due to its essential anti-ferroptotic function in the brain. Consumption of a high-fat, high-sucrose (HFHS) diet was reported to cause a decrease in *Gpx4* gene expression, however, which may exacerbate the oxidative damage and inflammation that results from an HFHS diet [122]. The ER-resident SELENOM may play an important neuroprotective role by regulating Ca^2+^ homeostasis and limiting ER stress [64]. An interesting parallel exists in that a study by Pitts et al. demonstrated that whole-body *Selenom* KO caused obesity, accompanied by leptin resistance, in mice [28]. Impaired hypothalamic function contributes to this phenotype as SELENOM was subsequently shown to enhance hypothalamic leptin signaling [123]. The underlying mechanism involves SELENOM suppressing ER stress through its intrinsic TXN-like activity.

The thyroid hormone regulator DIO2 is another selenoprotein with a well-documented role in hypothalamic physiology. Lacking expression of DIO1, the brain depends on DIO2 as the sole thyroid hormone activating enzyme [124]. The ME is lined with a special ciliated type of cells called tanycytes that regulate the exchange of hormones and nutrients between the ARC and the bloodstream. Tanycytes express high levels of DIO2, which become upregulated under inflammatory conditions, and may play an initiating role in hypothalamic thyroid hormone activation [125,126,127,128]. This possibility is supported by work from Coppola and colleagues showing that fasting upregulates DIO2 expression to elevate T3 levels in the ARC [129]. The increase in T3 then activates uncoupling protein-2 (UCP2) within AGRP neurons, resulting in elevated activity. Thus, DIO2 plays a role in promoting food seeking behavior through its metabolism of the thyroid hormone within the hypothalamus.

In 2017, a study by Yagishita et al. addressed the general role of hypothalamic selenoproteins using two mouse models with conditional KO of the *Trsp* gene [130]. Mice with rat-insulin-promoter-driven Cre (RIP-Cre) were crossed with mice in which the *Trsp* gene was flanked by lox-p sites, effectively ablating selenoprotein synthesis in the hypothalamus using the Cre-lox system. Since RIP is also expressed in pancreatic β cells, the researchers also crossed the *Trsp* floxed mice with insulin-induced gene 1 (Ins1)-Cre mice to stop the production of selenoproteins, specifically in β cells, but not the hypothalamus. In comparing the metabolic phenotypes of these two mouse models, the authors were able to deduce the contributions of hypothalamic selenoproteins. The *Trsp^RIP^KO* mice gained more excess weight, while on an HFD, compared to Trsp floxed control mice lacking Cre recombinase, and displayed glucose intolerance, hypothalamic leptin resistance, and systemic insulin resistance [130]. The *Trsp^Ins1^KO* mice did not display the same phenotype, so the effects were attributed to the loss of *Trsp* in the hypothalamus.

Yagishita and colleagues next assessed oxidative stress in the hypothalamus by imaging malondialdehyde (MDA), a product of polyunsaturated fatty acid peroxidation, as well as oxidized GSH (GSSG) and reduced GSH, and measuring lipid metabolites using liquid chromatography [131]. Mice with deletion of *Trsp* in the hypothalamus had more MDA-positive cells and a higher GSSG/GSH ratio than controls with the largest differences observed in the PVN and the mediobasal hypothalamus (MBH), which contains the ARC. The hypothalamus of *Trsp^RIP^KO* mice also had increased levels of oxidized phosphatidylcholines. Further analysis determined that broad inhibition of selenoprotein synthesis caused oxidative damage to leptin receptor (LEPR)-expressing POMC neurons, which the authors hypothesized was a key factor in the development of leptin and insulin resistance. The authors proposed that a lack of POMC neuron activation by leptin and insulin downregulated the sympathetic tone in brown adipose tissue (BAT) to promote obesity. This hypothesis was supported by an observed decrease in UCP1 expression in *Trsp^RIP^KO* mouse BAT [132]. Interestingly, Cre-dependent upregulation of *Nrf2* (nuclear factor erythroid 2-related factor 2) alleviated both the metabolic phenotype and the oxidative damage to POMC neurons. As a transcription factor, NRF2 regulates GSH metabolism-related genes, including *Gpx4*, and the authors concluded that increasing NRF2 in astrocytes upregulated GSH promoting genes and may have, subsequently, supplied GSH to neurons [133,134,135].

The study by Yagishita et al. provided strong evidence that selenoproteins are needed in the hypothalamus to maintain energy homeostasis and that disturbances in their activity can have significant deleterious metabolic effects throughout the body. A key aspect is that the RIP-Cre driver affected multiple neuronal types beyond just POMC neurons, as well as astrocytes in the hypothalamus [130]. The conditional *Trsp* knockout was confirmed to not have affected AGRP neurons, however. As mentioned above, AGRP neurons are unique in that they are the most active in detecting homeostatic signals due to their partial ability to bypass the BBB. There is also evidence that this influential population of neurons develops leptin resistance prior to the rest of the hypothalamus during obesity [104]. As such, AGRP neurons have gained increasing attention from researchers as candidate targets for the treatment of metabolic disorders [103,120,136]. The role of selenoproteins, specifically in AGRP neurons, was more recently addressed in a study that used a conditional KO of *Trsp* using AGRP promoter-driven Cre expression.

Ablation of selenoprotein synthesis just in AGRP neurons surprisingly resulted in the opposite phenotype as that of *Trsp^RIP^KO* mice, as *Trsp^AGRP^KO* mice gained less weight on an HFD than controls [35]. This effect was only observed in female *Trsp^AGRP^KO* mice, however, as male mice were unaffected by AGRP-specific *Trsp* deletion. Although the underlying cause of the sex-dependent nature of this phenotype was not determined, it is not all that surprising, as the hypothalamus is a very sexually dimorphic brain region, especially in terms of the regulation of energy metabolism [137,138,139]. Interestingly, female *Trsp^AGRP^KO* mice did not develop leptin resistance, despite being fed an HFD, and seemed to have greater BAT activation than HFD-fed control mice [35]. Thus, like the study by Yagishita et al., the underlying mechanisms may have involved changes in the sympathetic control of BAT thermogenesis. Recently, an axis through which leptin inhibits AGRP neurons to upregulate sympathetic innervation of white adipose tissue (WAT) and BAT was discovered [140] and may be involved in these studies targeting AGRP neurons. The contrasting data gained from RIP-Cre and AGRP-Cre driven KO of selenoprotein translation demonstrated that selenium can dramatically impact whole-body metabolism by acting in the hypothalamus and that the nature of this relationship is cell type-dependent.

Yet another study that focused on AGRP neurons descended from the initial discovery of the impact of a selenium metabolism enzyme on energy balance. Roughly a decade ago, Seale and colleagues reported that global KO of the gene for the intracellular recycling enzyme selenocysteine lyase (*Scly*) resulted in metabolic deficits in mice [27]. By catalyzing the breakdown of selenocysteine residues from degraded selenoproteins into selenide, *Scly* allows the cell to re-use the selenide for de novo selenoprotein synthesis [141]. The *Scly* KO mice were noted, by Seale et al., to exhibit glucose intolerance, high levels of insulin and leptin in the serum, and hepatic steatosis, among other symptoms. When challenged with a selenium-deficient diet, the *Scly* KO mice developed metabolic syndrome, including obesity and hypercholesterolemia [27]. Interestingly, the results were sex-specific, as the phenotype was found to be milder in female mice. The authors rationalized that the disruptions to energy homeostasis caused by the loss of *Scly* may be due to insufficient selenoprotein action to prevent oxidative stress in one or multiple tissues. It was further hypothesized that increased oxidative stress in the liver, due to the absence of *Scly*, may promote the inhibition of insulin signaling by protein tyrosine phosphatase 1B, and, subsequently, reducing glucose uptake and lipogenesis.

Subsequent investigations by the same group of researchers led to the discovery that male *Scly* KO mice were significantly more vulnerable to developing HFD-induced obesity, and western blot analysis of hypothalamic tissue revealed that the expression of several selenoproteins in the hypothalamus decreased, due to a lack of *Scly* [142,143]. To pinpoint the underlying cause of the obesogenic phenotype induced by constitutive *Scly* KO, a conditional *Scly* KO in AGRP neurons was generated. Similar to the result obtained with AGRP-specific *Trsp* ablation, *Scly^AGRP^KO* mice were found to be less susceptible to HFD-induced obesity and maintained hypothalamic leptin sensitivity while on an HFD [34]. Unlike the *Trsp^AGRP^KO* mice, however, the metabolic phenotype of *Scly^AGRP^KO* mice was not sex-specific.

While the loss of *Scly* agitates selenoprotein expression, KO of *Trsp* prevents selenoprotein synthesis altogether and, therefore, likely causes a greater amount of oxidative insult. It is, therefore, possible that the AGRP neurons of *Scly^AGRP^KO* mice experience enough oxidative stress to alter their physiology, whereas in *Trsp^AGRP^KO* mice, the neurons are degenerating. Indeed, the phenotype of *Trsp^AGRP^KO* mice mirrors that of a model from another study in which progressive neurodegeneration was induced in AGRP neurons. Xu et al. deleted mitochondrial transcription factor A (TFAM) in AGRP neurons and observed that, while the males were largely unaffected, female *Tfam^AGRP^KO* mice had reduced adiposity [144]. This finding was initially surprising to the authors since chemical ablation of AGRP neurons in adult mice caused anorexia and death due to failure to thrive [145]. It was later revealed, however, that progressive AGRP neuron degeneration induced a compensatory mechanism of adult neurogenesis that produced new leptin-responsive AGRP neurons [146].

An alternative explanation for the lean phenotype caused by disrupting selenoproteins in AGRP neurons is that an increase in ROS simply causes the neurons to become less active. This hypothesis is supported by past experiments using patch clamp electrophysiology on mouse brain slices, which showed that ROS had an inhibitory effect on AGRP neuron activity, while stimulating POMC neurons [147,148]. Diano et al. hypothesized that peroxisomes, organelles involved in controlling ROS, actively work to maintain AGRP neuron activity and limit POMC neuron firing by reducing endogenously produced ROS levels [147,149,150]. Interestingly, multiple studies indicated that selenium shows an ability to promote antioxidant mechanisms by activating the transcription factors peroxisome proliferator-activated receptor alpha and gamma (PPAR-α/γ) [14]. One possible mechanism of action, through which a loss of selenoprotein function could inhibit AGRP neurons, involves hydrogen peroxide activation of ATP-sensitive potassium channels (K_ATP_ channels) [151,152,153]. The opening of K_ATP_ channels causes an efflux of potassium that hyperpolarizes the neuronal cell membrane [154]. The important role of K_ATP_ channels in AGRP neurons has already been established and is known to be involved in the mechanisms of action of some homeostatic hormones [155,156,157]. Furthermore, K_ATP_ channels have been suggested as a therapeutic target for treating metabolic disorders [158].

The fact that consumption of an HFHS diet is known to cause a decrease in hypothalamic GPX4 levels raised the question as to whether GPX4 is an essential enzyme in the hypothalamus. This question was addressed in a study by Schriever et al. that sought to determine the effects of conditional *Gpx4* KO in the hypothalamus [159]. In this study, the authors first confirmed that an HFHS diet reduced *Gpx4* expression in the hypothalamus and, then, generated both AGRP and POMC neuron-specific GPX4 KO mice using the Cre–lox system. Surprisingly, the loss of *Gpx4* in POMC neurons had no discernible effect on whole-body metabolism. Male *Gpx4^AGRP^KO* mice, on the other hand, gained more body weight and fat mass than controls while on an HFHS diet. Although male *Gpx4^AGRP^KO* mice exhibited reduced locomotor activity and respiratory quotient during the light phase, no changes were seen in either food intake or energy expenditure. Like the *Gpx4^POMC^KO* mice, however, female *Gpx4^AGRP^KO* mice showed no signs of altered metabolism, compared to controls, while on an HFHS diet. These findings contrasted with the observation that fasting lowers *Gpx4* expression in POMC neurons, but not AGRP neurons. Additionally, neither conditional KO model affected systemic glucose tolerance or the density of AGRP and POMC neurons. The mild impact on energy homeostasis and lack of any signs of neuronal degeneration suggested that, contrary to expectations, GPX4 antioxidant activity was not essential within AGRP or POMC neurons. These cell populations may depend more on other selenoproteins, such as those that reside in the ER, for which mounting evidence points to an essential role in the brain and metabolic disease [160,161].

To date, the majority of evidence that selenium is important for hypothalamic function is centered around the signal detection capabilities of the ARC, especially regarding leptin sensitivity [21]. Although GPX4 was surprisingly found not to be an essential player in maintaining ARC neuronal health and physiology, further investigations might uncover unique roles for other selenoproteins in the hypothalamus. One area with potential promise is adult hypothalamic neurogenesis. Although this phenomenon is not as well-characterized as neurogenic activity in other brain regions, like the hippocampus, hypothalamic neurogenesis impacts homeostatic processes, including energy metabolism, sexual activity, and temperature regulation [162]. Recently, a ground-breaking study delineated a central role for selenium in regulating exercise-induced hippocampal neurogenesis. In this study, Leiter et al. showed that liver-derived SELENOP is secreted into the bloodstream in response to physical exercise and binds to LRP8 in the hippocampus to stimulate neural precursor cells [93]. Interestingly, genetic disruption of either *Selenop* or *Lrp8* abolished the ability of exercise to induce adult hippocampal neurogenesis. Might SELENOP also play a similar role in mediating neurogenesis in the hypothalamus? Interestingly, circulating SELENOP is studied as a potential mediator of metabolic disorder and, although mechanisms involving the pancreas and BAT were reported, the physiological effects of SELENOP in the hypothalamus were not reported on [163,164,165]. Another potential area of interest is the intersection between selenium and the sexual dimorphism of the hypothalamus. Many studies on selenium biology yielded sex-specific results, including studies of energy metabolism [166]. Considering the numerous sex differences reported in the studies reviewed in this section, could hypothalamic function be an underlying mechanism to explain the sexually dimorphic effects of selenium and selenoproteins on energy homeostasis? The role of selenium in hypothalamic function is a growing field and further investigation into these areas is warranted.

## 4. Signals from Brain to Body

**Figure 2:**
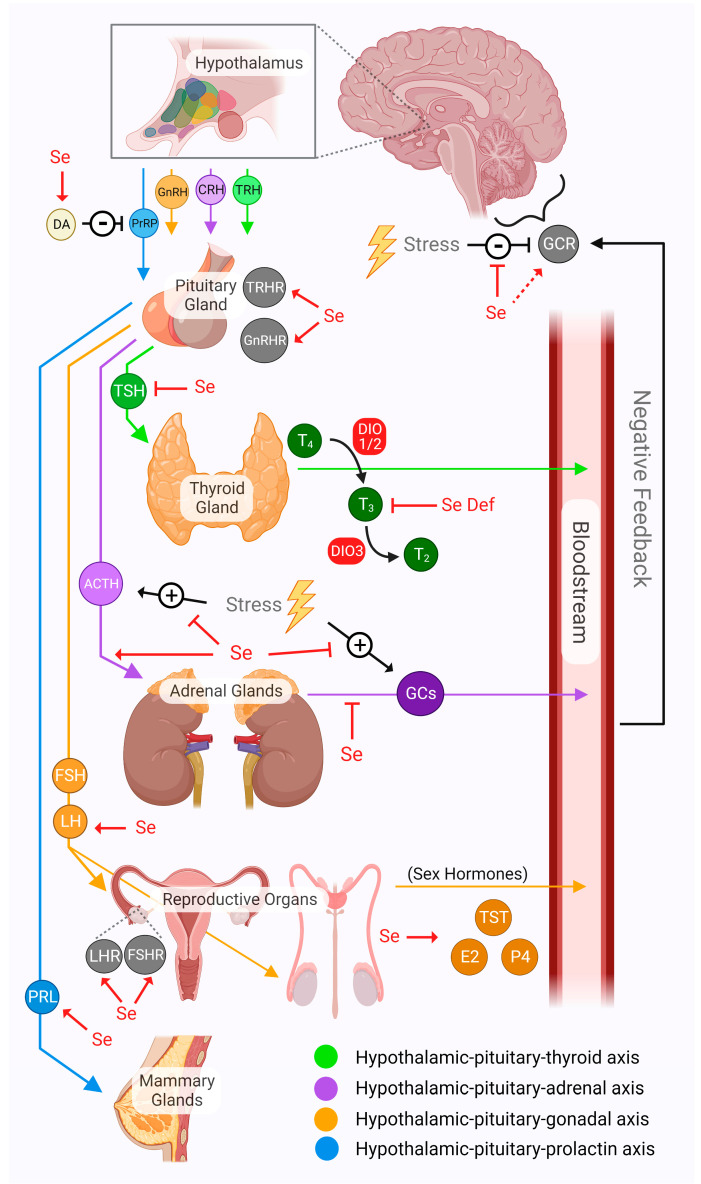
Hypothalamic axes of communication and the various effects of selenium. The hypothalamus sends regulatory signals throughout the body by controlling the release of hormones from the anterior pituitary gland. Selenium (Se) has shown an ability to upregulate the thyrotropin-releasing hormone receptor (THR) and gonadotropin-releasing hormone receptor (GnRHR) in the pituitary gland. Along the hypothalamic–pituitary–thyroid (HPT) axis, selenium has shown a capacity to limit thyroid-stimulating hormone (TSH) from the pituitary gland and is intimately involved in thyroid hormone metabolism, as it is used to synthesize the iodothyronine deiodinase (DIO) sub-family of selenoproteins. Evidence from multiple lines of investigation suggest that selenium plays a role in normalizing the HP–adrenal axis under conditions of elevated stress, through mechanisms involving the limiting of adrenocorticotropic hormone (ACTH) secretion from the pituitary and glucocorticoid (GC) secretion from the adrenal gland. Additionally, the negative feedback loop of the HPA axis is supported, by helping the brain maintain GC receptor (GCR) expression levels, which are typically downregulated by chronic over-activation. Selenium seems to promote the secretion of luteinizing hormone (LH) from the pituitary gland and the expression of the receptors for LH (LHR) and follicle-stimulating hormone (FSHR) in the ovaries. Selenium is also important for maintaining sufficient production of sex hormones, including testosterone (TST), estradiol (E2), and progesterone (P4). Finally, selenium affects the HP–pituitary axis by supporting prolactin (PRL) secretion from the pituitary. There is also potential for selenium to regulate the hypothalamic release of prolactin-releasing peptide (PrRP), through its influence on dopamine (DA) transmission. CRH: corticotropin-releasing hormone, T4/3/2: thyroid hormones. Effects of selenium are shown in red.

The hypothalamus is responsible for regulating homeostasis throughout the body and does so through multiple axes of communication. For example, neurons of the hypothalamus have connections to brain stem nuclei that control different organs via direct sympathetic nervous innervation. The hypothalamus also releases neurohormones that enter the bloodstream to bind receptors in peripheral tissues, or can act on the anterior pituitary to induce the release of various pituitary gland hormones that have specific targets in the body. This review mainly considers the endocrine pathways controlled by the hypothalamus. The axes regulating the thyroid and adrenal glands are the most well-characterized in terms of interactions with selenium, but evidence relevant to other axes is also discussed. The effects discussed in this section are graphically summarized in Figure 2.

### 4.1. Hypothalamic–Pituitary–Thyroid Axis

The most well-characterized pathway of communication from the hypothalamus in terms if interactions with selenium is the hypothalamic–pituitary–thyroid (HPT) axis. Thyrotropin-releasing hormone (TRH), originating from the hypothalamus, binds its receptor in the anterior pituitary to cause the release of thyroid-stimulating hormone (TSH), which goes on to induce thyroid hormone secretion from the thyroid gland [167]. Activation of the HPT axis has a direct impact on energy homeostasis and an imbalance within the axis can make an organism more vulnerable to developing metabolic disorders and obesity [168]. Among the major physiological processes affected by thyroid hormones are energy expenditure, thermogenesis, liver metabolism, bone homeostasis, and cardiovascular function [169,170,171,172,173,174]. Selenium plays an essential role in the HPT axis as the DIO selenoprotein sub-family is responsible for the activation and de-activation of thyroid hormones via deiodination [175,176].

Many reviews have been published in recent years summarizing the ways in which selenium is implicated in numerous pathologies related to thyroid endocrine abnormalities, including autoimmune thyroiditis [177,178], Hashimoto’s thyroiditis [179,180], Graves’ disease [181,182] and Graves orbitopathy [181], subclinical hypothyroidism [183], subclinical hyperthyroidism [184], goiter [185], postpartum thyroid dysfunction [186], post-COVID-19 thyroid dysfunction [187], as well as thyroid disorders in general [188]. Reviews have also been published highlighting the relevance of selenium to non-thyroidal conditions that remain metabolically pertinent, including metabolic syndrome [189], type 2 diabetes [190], gestational diabetes mellitus [191], and gestational disorders [192]. Despite numerous studies [178] reporting that selenium supplementation reduces thyroid autoantibody levels in patients with autoimmune thyroiditis, however, recent meta-analyses have found evidence of clinical efficacy to be lacking [193,194,195]. Numerous reviews have also been published summarizing the role of selenium in endocrinological disease [196], thyroid function [72,197,198], pathophysiology [198], and maintaining homeostasis in general [199]. As such, the focus of this sub-section is on articles published within the last year, to provide the latest insights from this well-investigated field. An emphasis was placed on publications describing how selenium availability affects the release and metabolism of the following hormones, relevant to the HPT axis, as well as their receptors; TRH, TSH, and the thyroid hormones (T4, T3, T2).

The environmental pollutant di-(2-ethylhexyl) phthalate (DEHP) is known to disrupt thyroid hormone function [200]. The relationship between selenium and DEHP was first formally discussed in literature in 1991 [201]. More recently, both selenium and selenium nanoparticles were found to be protective against the toxic effects of DEHP. Selenium was found to ameliorate the effects of DEHP exposure, both in a rat model of DEHP thyrotoxicity and in an immortalized human thyroid cell line [202]. These protective effects included maintaining TRH receptor abundance in the pituitary at both the protein and transcript levels. Additionally, selenium nanoparticles exerted protective effects against DEHP-induced thyroidal disruption in male rats, including by maintaining levels of free T3 (fT3) and T4 (fT4) in the serum [203]. The heavy metal nickel chloride was another toxic pollutant that induced ROS accumulation [204]. The effects of selenium on nickel chloride induced hepatic lipid peroxidation were first investigated in 1998 [205]. Recent reports indicated that selenium was protective against nickel chloride-induced reductions in plasma T3 and T4, and increased in TSH in pregnant rats [206]. It is important to note that some of these studies made use of selenium nanoparticles, which are composed of selenium particles bound to various active compounds to improve their effectiveness [207]. Such compounds allow for efficient delivery of selenium to cells while avoiding the risk of toxicity involved with supplementing with other forms of selenium [208]. Selenium nanoparticles gained attention for their cytoprotective effects in neurons and astrocytes [209], and in their ability to cross the BBB [210].

Several studies were recently published reporting on selenium status and hormone levels in pregnancy. Contrasting evidence on the association between serum selenium status and circulating levels of T3 were reported, with some finding low serum selenium to be associated with reduced fT3 [191], and others finding low serum selenium to be associated with increased fT3 [211]. The association of low serum selenium with high fT3 was also found to correlate with high total T3. However, a lack of association was observed regarding T4 levels, suggesting this relationship was not mediated by thyroidal hormone production. Rather, the inverse association between plasma selenium levels and the total/free T3 ratio may have been communicated via levels of DIO3, the selenoprotein responsible for inactivating T3. Low serum selenium was also associated with an increased incidence of gestational diabetes mellitus [191]. A non-linear association was also found between third-trimester maternal selenium status and TSH levels, with a significant inverse relationship found at maternal serum selenium levels below 103.7 ug/L, but no significant relationship above that threshold [212].

Several associations have been established between an individual’s selenium status and HPT hormone levels. A recent study, following 22 Japanese patients at risk for selenium deficiency, found that inadequate selenium status was associated with abnormal thyroid hormone levels, where TSH, fT4, and fT4/fT3 ratio were low, and fT3 was high [213]. Interestingly, a recent animal study found that when mice were placed on a low selenium diet (0.02 mg/kg), selenium levels in the thyroid gland were maintained, but reduced selenoprotein expression was observed in the liver and kidneys, targets of thyroid hormones [214]. While selenium supplementation was reported to induce no significant therapeutic effect on patients with subclinical hypothyroidism [215], co-treatment of selenium was found to enhance the efficacy of levothyroxine sodium (LT4) in treating chronic lymphocytic thyroiditis patients with hypothyroidism, yielding greater improvements to inflammatory factors, compared to treatment with LT4 alone [216]. Selenium supplementation was also found to increase the efficacy of antithyroid drugs in the treatment of Graves’ disease [217].

In a rat model of experimental autoimmune thyroiditis, selenium yeast was found to partially attenuate immune imbalances, including preventing fluctuations in serum TSH [218]. Additionally, in a recent study following males with chronic autoimmune thyroiditis suffering from infertility, selenium supplementation was effective at improving sperm parameters [219]. Collectively, these findings highlight the breadth of research currently being conducted on selenium and the HPT axis. While most studies addressed the impact of selenium on thyroid hormone secretion and metabolism, there was also a reported effect on the TRH receptor.

### 4.2. Hypothalamic–Pituitary–Adrenal Axis

The hypothalamic–pituitary–adrenal (HPA) axis comprises the endocrine component of the “fight or flight” stress response [220,221]. In response to stressful stimuli, the hypothalamus releases corticotropin-releasing hormone (CRH), which induces the secretion of adrenocorticotropic hormone (ACTH) from the anterior pituitary. ACTH then acts upon the adrenal gland to cause the release of corticosteroids, including the glucocorticoid (GC) class of stress hormones. The GC receptor (GCR) is present in nearly every tissue in the body. Thus, the effects of GC action are wholistic, but are commonly known to include gluconeogenesis, arousal, and immunity [222,223,224]. Due to their anti-inflammatory properties, GCs are widely prescribed for various conditions and diseases [225]. Selenium seems to play a strong role in protecting against the damage and dysfunction caused by overactivation of the HPA axis. This relationship has been studied in the brain more intensively in recent years, using rodent models, and progress in the field was recently reviewed by our group [226]. In sum, treatment with selenium, mostly in the form of selenium-containing compounds, was shown to have a therapeutic effect against stress-induced neurological impairments and oxidative damage in rodent models.

Over the past couple of years, three additional studies were published demonstrating the promising capabilities of seleno-compounds. The first, by Muller et al., reported that m-trifluoromethyl-diphenyl diselenide [(m-CF_3_-PhSe)_2_] exerted antidepressant-like effects in a mouse model of lifestyle-induced depression [227]. This model relied on a combination of a calorically dense diet and ethanol consumption to induce a depressive-like phenotype in mice. This phenotype was associated with an increase in opioid receptor levels and a decrease in GCR levels in the cerebral cortex [227]. These molecular changes, as well as the associated depressive-like behavioral phenotype, were abolished by (m-CF_3_-PhSe)_2_ treatment [227]. Treatment also lowered levels of the oxidative marker MDA, while upregulating the antioxidant enzyme superoxide dismutase (SOD) in the cerebral cortex. In addition to its pharmacological effects and ability to support SOD expression, previous work from the same group confirmed that (m-CF_3_-PhSe)_2_ had its own endogenous antioxidant activity [228].

Another compound, 7-chloro-4-(phenylsenyl) quinoline (4-PSQ), was also recently shown by de Oliveira and colleagues to be effective in attenuating depressive-like phenotypes in mice following acute restraint stress (ARS) [229]. Among other mechanisms, the researchers reported that direct attenuation of the HPA axis activation may be involved in this finding, as 4-PSQ treatment prior to ARS maintained circulating corticosterone (CORT), the main active GC in mice, at levels similar to those of the unstressed controls [229]. This suggested that prophylactic administration of 4-PSQ might have the capacity to prevent hyperactivation of the HPA axis, keeping CORT levels near baseline. Indeed, similar results from previous studies suggested that selenium had a “normalizing” effect on the HPA axis under stressful conditions and prevented the elevation of circulating GC levels usually caused by stress. This effect was typically accompanied by a normalization of ACTH levels as well, suggesting that selenium regulated the HPA axis at the level of the hypothalamus or pituitary gland. One common theme noted in various papers that reported an HPA axis normalization effect was that selenium-based therapy reversed the GCR downregulation typically caused in the brain by chronic stress [226]. Brain-residing GCR provided the negative feedback loop that prevented hyperactivity of the HPA axis. and its downregulation contributed to the dysfunction of the axis [230]. The restoration of GCR expression on the part of selenium was observed in the prefrontal cortex and the hippocampus, but it remains to be tested on GCR in the hypothalamus, which is the most influential site in terms of maintaining the negative feedback loop.

The third recent study utilized the organoselenium compound, 3-[(4-chlorophenyl)selanyl]-1-methyl-1*H*-indole (CMI), which was previously reported to ameliorate depressive-like symptoms in mice following ARS [231,232]. To investigate the functions of CMI further, Casaril et al. used a mouse model in which chronic intragastric (i.g.) CORT administration caused mice to develop anhedonic- and anxiogenic-like symptoms [233]. This model of stress mimicked long-term hyperactivation of the HPA axis, which had been shown to illicit depressive-like phenotypes paralleled in humans [234]. In the study by Casaril et al., a single i.g. dose of CMI following two weeks of CORT ingestion alleviated the behavioral deficits and reduced ROS and lipid peroxidation, while normalizing the expression of *Gcr*, brain-derived neurotrophic factor (*Bdnf*), synaptophysin (*Syp*) and *Nrf2* in the hippocampus [233]. Furthermore, this therapeutic effect was abolished following co-administration of PI3K and/or mTOR inhibitors (LY294002 and rapamycin) via intracerebroventricular (i.c.v.) injection. This suggested that the mechanism of action of CMI on stress-induced depressive-like phenotypes might rely partly on PI3K/mTOR signaling pathways within the brain. The authors proposed that CMI upregulated *Bdnf* in order to activate the PI3K/mTOR pathway and, subsequently, upregulated *Syp*, which was reduced by CORT administration alone. Thus, a restoration of synaptogenesis could play a central role in the fast-acting ability of CMI to reverse the effects of chronic CORT.

Similar to previous studies using organoselenium agents, CMI treatment reduced plasma CORT back to the level of controls. Plasma CORT was not measured in the experiments with LY294002 and rapamycin, however, so it is still unclear whether the PI3K/mTOR pathway is involved in a stabilization of the HPA axis by CMI. Interestingly, the PPAR-γ agonist rosiglitazone was shown to reverse the HPA axis over-activation present in diabetic rats by upregulating PI3K [235]. Moreover, the hypothalamus is an active center of adult neurogenesis and synaptogenesis [236]. Could these processes be involved in normalizing the HPA axis in a way that is facilitated by selenium? As mentioned in the section on selenium and hypothalamic function, a role for SELENOP and selenium in hippocampal neurogenesis was recently uncovered [93].

Several studies have also been published on the effects of selenium on GCs in fish. Like in mammals, GCs are a major stress hormone in fish and are secreted by the hypothalamic–pituitary–inter-renal (HPI) axis, analogous to the HPA axis in mammals [237,238]. One study found that treatment of iridescent sharks (*Pangasianodon hypothalamus*) with selenium nanoparticles effectively protected against raised cortisol levels following arsenic and heat stress [239]. A contrasting result was found in gilthead seabream (*Sparusaurata*), where long-term supplementation with hydroxy-selenomethionine (OH-SeMet) resulted in elevated plasma cortisol levels following a crowding stress test [240]. Basal cortisol levels were unaffected by supplementation, suggesting that OH–SeMet supplementation may have sensitized the HPI stress response. Another study in white sturgeon (*Acipenser transmontanus*) found that exposure to environmental SeMet decreased tissue sensitivity to glucocorticoids, as inferred by a lower abundance of GCRs [241].

In vitro studies from Miller et al., on the effects of selenite and SeMet on adrenocortical cell lines isolated from rainbow trout (*Oncorhynchus mykiss*) and brook trout (*Salvelinus fontinalis*), with a relevant focus on ACTH-stimulated cortisol secretion, were conducted [242]. Researchers found that cortisol secretion in these cell lines was impaired by selenite but not by SeMet. Their findings suggested that this impairment was caused by a disruption of the cortisol biosynthetic pathway, leading the investigators to attempt to restore cortisol synthesis by introducing precursors and signaling molecules in a step-wise manner to isolate the disrupted step. In brook trout cells, cortisol secretion was restored by pregnenolone, suggesting that selenite impaired cortisol secretion in this cell line by disrupting pregnanolone synthesis. Pregnenolone is synthesized from cholesterol [243], and not only does selenium have a documented hypocholesterolemic effect, but the SELENOP receptor in the brain, LRP8, is heavily involved in cholesterol homeostasis, pointing towards a potential avenue for further investigation [244,245]. In the same study by Miller et al. on rainbow trout cells, cortisol secretion was restored by N^6^,2′-o-dibutyryladenosine 3′,5′-cyclic monophosphate (dbcAMP) [242]. This suggested that selenite might disrupt cortisol secretion in this cell line early on in the pathway by interfering with the binding of ACTH to melanocortin 2 receptors, resulting in disrupted cAMP production via adenylyl cyclase.

Additional insight on the potential role of selenium action in the adrenal gland is provided by a study on pigs that found that selenium deficiency reduced antioxidant capacity and induced oxidative stress in adrenal tissue, with evidence that this effect was mediated by the toll-like receptor 4 (TLR4)/NF-kB pathway [246]. Specifically, investigators found that Se deficiency was associated with reductions in miR-30d-R_1, a microRNA (miRNA) that negatively regulates TLR4 expression, suggesting that the observed inflammation might have been caused by dysregulation of the TLR4/NF-kB pathway [247]. Interestingly, TLR4 over-expression in human adrenocortical cells inhibited cortisol and aldosterone production [248]. Selenium may, therefore, support the HPA axis by facilitating a mechanism of miRNA downregulation of TLR4 to promote adrenal steroidogenesis. Yet another study on mice. by Chanoine et al., revealed that selenium deficiency blunted the adrenal response to ACTH, resulting in decreased CORT secretion [249]. The authors also observed an increase in adrenal isoprostane F_2α_, which was indicative of oxidative stress, and a decrease in adrenal gland GPX activity, which might have contributed to the blunting of the ACTH response.

Yet another interesting relationship between selenium and the stress response is the role that selenoproteins play in the development of the HPA axis. Activation of the *Selenot* gene was seen during neuroendocrine cell differentiation in the adrenal medulla [250]. After the binding of pituitary adenylate cyclase-activating peptide (PACAP) to its receptor, this triggered the pathways involved in neurogenesis and *Selenot* transcription using the NRF-1 transcription factor and PPAR-γ coactivator 1α (PGC-1α). This led to production of SELENOT to combat oxidative stress during cell differentiation that would otherwise hinder the development of the neuroendocrine system [250]. Conversely, GCs were shown to have an ability to alter selenoprotein expression in various tissues, including the hypothalamus. Gene expression of both *Selenop* and *Selenos* were affected by GCs in human embryonic kidney (HEK-293) cells and differentiated human adipocyte-like cells, respectively [251,252]. Using a model of GC-induced metabolic impairment, in which mice were administered CORT via drinking water for 4 weeks, Wray et al. discovered an effect on several genes in the hypothalamus [253]. RNA-seq analysis of ARC samples from obese CORT-administered mice revealed a downregulation of *Scly*, and an upregulation of both *Selenop* and *Dio2*. Targeted deletion of *Dio2* in the mediobasal hypothalamus did not prevent the metabolic effects of CORT, which included over-eating, excess weight gain, and glucose intolerance. The effects on *Scly* may warrant further investigation, however, as the metabolic deficits induced by chronic GC consumption bear similarities to the impairments observed in mice with constitutive *Scly* KO [27,142].

Clinical research provides further hints at interactions between selenium and GCs in humans. A study by Marano et al. found that serum selenium levels increased in patients treated with CORT [254]. These results suggested that selenium might be retained in the body, or, perhaps, become more mobilized, in response to long-term HPA axis activation. In another clinical study, supplementation with selenium increased adrenocortical function in patients taking GCs for treatment of various diseases [255]. These findings implied the possibility that the optimal amount of selenium intake may be higher for patients undergoing long-term GC therapy, or for individuals experiencing chronic stress. Although this is purely hypothetical, continued investigation of both animal models and humans could elucidate whether dietary selenium requirements might change during conditions of HPA axis dysfunction.

### 4.3. Hypothalamic–Pituitary–Gonadal Axis

The endocrine target tissues of the hypothalamic–pituitary–gonadal (HPG) axis are the gonads; the testes in males and ovaries in females. The biochemical cascade is initiated by hypothalamic release of gonadotropin-releasing hormone (GnRH) which stimulates the secretion of gonadotropins, namely, luteinizing hormone (LH) and follicle-stimulating hormone (FSH), from pituitary gonadotrophs, which then go on to trigger the release of sex hormones from the gonads [256]. Testosterone is the main hormone released by the testes, which are also capable of secreting activin, inhibin, and insulin-like-growth factor 3 (IGF-3), as well as anti-Müllerian hormone and estradiol, albeit to a lesser extent compared to the ovaries. Estrogens, progesterone, and inhibin are released by the ovaries, as well as small amounts of testosterone [256]. These hormones are able to act on the hypothalamus to inhibit the release of GnRH [256,257], completing the negative feedback loop. The HPG axis is largely involved in regulating reproduction and development, but also affects immunity, neurological function, and cardiovascular health, and helps shape the sexual dimorphism of the brain [258,259,260,261,262].

There are currently no reported data describing a direct effect of selenium on GnRH; however, there is potentially an interaction with LH. Selenium administration was shown, by Kheradmand et al., to reverse the ability of the synthetic glucocorticoid dexamethasone to lower serum LH levels [263]. Although this could result from selenium antagonizing the cellular effects of dexamethasone by reducing ROS, selenium treatment alone caused an upward trend in serum LH suggesting the possibility that selenium may promote LH production or release its own. In support of this hypothesis, other researchers proposed that selenium can upregulate GnRH receptor (GnRHR) activity in the anterior pituitary to increase LH synthesis [264].

Most of the available evidence on the potential for selenium to support the HPG axis exists on the level of sex hormone production. While inverse correlations were found between plasma selenium and testosterone levels in cohorts of male endurance runners [265] and women with polycystic ovary syndrome (PCOS) [266], randomized control studies reported no significant effects of selenium supplementation on testosterone levels [267,268]. A study utilizing rats, however, found that selenium depletion lowered testosterone levels while decreasing testicular mass and adversely affecting testicular morphology [269]. Although the mass of the testes of the first generation of selenium-deficient rats was not significantly changed, each successive generation showed increasingly worsening symptoms. Interestingly, the administration of either GnRH or LH induced a smaller amount of testosterone secretion in selenium deficient rats, implying that testosterone production might be impaired or, perhaps, there is a disruption of the receptors or signaling pathways for GnRH and LH, and the testicular phenotype cannot be explained solely by a decrease in GnRH or LH secretion. Indeed, selenium was shown to support testosterone synthesis by activating the extracellular signal-regulated kinase (ERK) signaling pathway in sheep Leydig cells [270].

The importance of selenium to male fertility is well-documented and is largely due to the role of selenoproteins in antioxidant defense, and the vulnerability of the testes, and subsequently, sperm fertility, to oxidative insult [271]. In fact, the testes require a large amount of selenium and are in competition with other organs when selenium supply is low [272]. However, the benefits of selenium supplementation on fertility may vary depending on baseline selenium status. A study following a cohort of Iranian men presenting with infertility, due to oligospermia, asthenospermia, or teratospermia, found that selenium supplementation (200 ug/day) improved sperm characteristics and modulated serum hormone levels [273]. Specifically, selenium supplementation increased total sperm count, sperm concentration, sperm motility, ejaculate volume, and strict morphology in this cohort [273]. Contrastingly, a study following a cohort of North American men with no known fertility issues reported no change to sperm quality following 300 ug/day of selenium supplementation [274].

Animal studies have revealed the numerous ways selenium supports reproduction through male fertility. Perhaps most notably, an enzymatically inert GPX4 isoform acts as a structural component of spermatozoa and is indispensable for sperm motility [275]. Additionally, selenium plays a largely protective role through redox regulation and has been shown to attenuate reproductive impairment in males of various species following exposure to many different injurious compounds, including DEHP [276], di-n-butyl phthalate (DBP) [277], diclofenac [278], lead [279], ciprofloxacin [280], mercury [281], and dexamethasone [263]. In the cases of DEHP, DBP, diclofenac, and lead exposure in rats, selenium similarly mitigated testicular injury by maintaining testosterone, LH, and FSH levels. Selenium treatment also protected expression of IGF-3 following DBP exposure [277], and lowered blood and testicular lead levels following lead exposure [279]. In laying hens, selenium attenuated the effects of mercury exposure by elevating LH, FSH, progesterone, and estradiol levels closer to those of controls [281]. Ciprofloxacin treatment is associated with elevated ROS levels [282], which can induce reproductive dysfunction in males [280,282]. These deleterious effects include reductions in serum hormone levels, poor sperm quality, testicular impairment, depletion of GSH, inhibition of catalase, SOD, glutathione-S-transferase, and GPX, as well as elevation in nitric oxide levels and myeloperoxidase activity; all of which were observed to be ameliorated by selenium co-treatment [280]. Selenium treatment was also shown to protect against the deleterious effects of dexamethasone on male fertility and sperm parameters in mice [263]. Specifically, selenium treatment was associated with elevated expression of *Catsper-1* and *Catsper*-*2*, genes involved in sperm motility [263]. This might implicate transcriptional regulation as part of the protective mechanism of selenium.

In a study on ungulates, ewes and goats with estrogen-dominant reproductive disorders were found to have high serum selenium, while levels were low in those with progesterone-dominant reproductive disorders or ovarian inactivity [283]. Selenium supplementation was also found to affect progesterone levels of cows and sheep across different phases of estrus [284,285] and gestation [286,287]. Further investigation into relevant mRNA transcript levels in the corpus luteum uncovered no change in transcripts involved in progesterone synthesis, but yielded an increase in transcripts (*Ldlr* and *Hsl*) involved in the regulation of cholesterol availability [288]. This might suggest that selenium-induced effects on progesterone production in the corpus luteum are consequences of changes in cholesterol availability, rather than directly influencing progesterone synthesis. One interesting clue lies in the fact that LRP8, the receptor that binds SELENOP for selenium delivery, also plays an important role in cholesterol homeostasis [245,289].

Exposure to selenium containing compounds was also shown to affect the HPG axes of fish. In female zebrafish, exposure to environmentally relevant levels of selenium, in the form of sodium selenite, resulted in transcriptional downregulation of the genes for the receptors for LH, *lhr*, and upregulation of the FSH receptor, *fshr*, in the ovaries, presumably affecting gonadal responsiveness to the pituitary gonadotropins [290]. In female rainbow trout, selenium exposure, in the form of dietary SeMet, stimulated vitellogenesis and increased levels of sex steroid hormones, as well as steroidogenic proteins and their transcripts [291]. Specifically, SeMet exposure resulted in higher levels of androstenedione, estrone, estradiol, and testosterone. Implicated steroidogenic protein transcripts included *pbr, P45scc*, and *3β-hsd* [291]. Therefore, SeMet, which is a highly abundant form of selenium in foods, may promote vitellogenesis via direct stimulation of ovarian tissue steroidogenesis [292].

The SECISBP2 protein is integral to the incorporation of selenocysteine residues into selenoproteins [293]. In an in vitro study using a human trophoblast cell line, knocking down SECISBP2 impaired the proliferative, migratory, and invasive abilities of the cells [294]. The cells also showed a reduction in β-HCG at both transcript and protein levels, as well as inhibited progesterone production [294]. PI3K/Akt and ERK signaling pathways were implicated via an associated downregulation in transcript levels [294]. In another in vitro study utilizing a buffalo oocyte cell line, cells treated with selenium had a significantly faster rate of nuclear maturation, and decreased germinal vesicle stage [295]. While selenium treatment was not associated with differences in cell development at any other meiotic stage, several genes involved in oocyte gene expression were affected. Differential expression was noted in the downregulation of *Casp3* and *Amh*, and the upregulation of *Gpx4* and *Sod* [295]. Selenium treatment of buffalo oocytes was also associated with a reduction in Pla2g3 transcript levels [295], a phospholipase involved in lipid metabolism [296]. A study conducted on primary luteinized granulosa cells cultured from the ovarian follicles of goats found that selenium treatment stimulated cell proliferation and increased markers related to estradiol production (3β-HSD, p-Akt, cAMP, and steroidogenic acute regulatory protein) [297]. Thus, selenium may primarily regulate the HPG axis by controlling sex hormone production through its influences on redox balance and lipid metabolism.

### 4.4. Hypothalamic–Pituitary–Prolactin Axis

The hypothalamic–pituitaryprolactin/mammary (HPP) axis regulates the release of prolactin from the anterior pituitary, after which the pleiotropic hormone acts widely throughout the body to stimulate a range of effects, including lactation [298]. Unlike other hypothalamic–pituitary axes, the HPP axis does not have a target endocrine tissue to provide negative feedback regulation of its release. Hypothalamic inhibition of prolactin release is instead regulated by dopamine, a catecholamine neurotransmitter, the release of which is stimulated by prolactin [298]. Due to its involvement in reproduction, studies conducted during estrus, gestation, or parturition typically report on prolactin, alongside other reproductive hormones, such as LH, FSH, and estradiol.

Selenium supplementation was shown to attenuate the reduction in reproductive hormones, including prolactin, associated with gestational lead exposure [299] and diabetes [300] in rats. Similarly, in beef steers, selenium supplementation, and specifically with organic, or a 1:1 mix of organic and inorganic, selenium, was shown to mitigate the decrease in serum prolactin that is characteristic of fescue toxicosis [301,302]. The authors suggested that this effect might involve dopamine regulation, as a reduction in dopamine type two receptor (*D2r*) mRNA was detected [302]. Additionally, while prolactin mRNA levels were higher in the pituitary cells of organic and mixed selenium treated steers, no change was detected in the abundance of pituitary transcription factor, *Pit-1* [302]. Since PIT-1 is responsible for stimulating expression of prolactin mRNA [303], a lack of change in regulatory protein abundance might suggest the involvement of epigenetic mechanisms in increasing prolactin expression. It is worth noting that selenium deficiency was shown to increase dopamine turnover in various brain regions and, thus, could affect the dopaminergic pathways responsible for regulating prolactin release [304,305,306,307].

While the molecular mechanisms of selenophosphate synthetase 1 (SEPHS1) have yet to be determined, it is thought to be involved in selenium metabolism and recycling [59,308,309] and is known to be vital for cell survival and proliferation [310]. Studies on *Sephs1* KO mice suggested an influence on prolactin dynamics. Although SEPHS2 is a selenoprotein, SEPHS1 does not contain a Sec residue in eukaryotes; however, it is nonetheless involved in redox homeostasis [311]. Knocking out *Sephs1* in mice was embryonically lethal by E9.5, due, in part, to heavily impaired organogenesis [310]. Transcriptomic analysis of *Sephs1* KO embryos saw upregulation of the genes involved in prolactin and IGF signaling pathways [310], suggesting that impaired selenium metabolism during development might disrupt prolactin signaling in ways that impair organogenesis. This result also implicated the hypothalamic–pituitary–somatotropic axis, which is discussed in the next section.

Selenium supply was demonstrated to affect hormonal profiles of female animals across various species. In rats, selenium supplementation increased plasma LH, FSH, estradiol, and progesterone in female adults during estrus [312]. Selenium supplementation was also found to affect systemic progesterone and prolactin in beef cows; increasing progesterone throughout gestation, as well as the early luteal phase of estrus, and decreasing prolactin in late lactation [285]. The maternal hormone profiles of ewes were susceptible to alterations in selenium supply during parturition, but not gestation [313]. In this study [313], ewes supplemented with high selenium (77 μg/kg body weight) had greater concentrations of estradiol-17β during parturition, compared to those receiving adequate selenium (11.5 μg/kg BW). It is not clear from these studies whether selenium exerts these effects by acting upon the HPP, as pituitary-derived prolactin can be stimulated by signals from other areas of the body, and prolactin can also be produced in other tissues, such as the placenta during pregnancy, but the correlations discussed here are a potential starting point for future studies involving selenium [314,315].

### 4.5. Hypothalamic–Pituitary–Somatotropic Axis

The hypothalamic–pituitary–somatotropic (HPS) axis involves the release of somatocrinin and somatostatin to either stimulate or inhibit, respectively, the release of growth hormone (GH) from the pituitary gland. There is some evidence that selenium can impact GH and IGF-1 signaling pathways in certain species of fowl and fish. The results are somewhat contradictory, however. In chickens, dietary selenium supplementation was found to upregulate growth-related genes, including genes for growth hormone receptor (*Ghr*) and *Igf-1*) [316]. In ducks, supplementation was associated with a reduction of serum IGF-1 [317]. In both studies, supplementation resulted in increased body weight gain [316,317]. Selenium containing compounds were found to have similar effects in fish, either increasing [318,319], or decreasing [320] *Ghr* and *Igf-1* transcript levels, depending on the species. While selenium supplementation was found to increase growth in some species of fish [318], it also impaired growth in others [319]. Notably, in rainbow trout, dietary selenium was associated with impaired growth, as well as an upregulation of genes involved in the GHR and IGF-1 signaling pathways [319]. This suggested a compensatory nature to these molecular changes, perhaps as a response to growth impairment.

Limited associations have been found between serum selenium and IGF-1 in humans. In a population of Italian participants aged 65 years or older, serum selenium and IGF-1 levels were found to be positively associated [321]. This association was also found in a study following North American women between the ages of 18 and 22 [322]. A review of the literature did yield any publications on the relationship between selenium status and GH protein or transcript levels in humans.

### 4.6. Oxytocin and Vasopressin

The hypothalamus synthesizes oxytocin and vasopressin, neurohormones that are heavily involved in the circuitry of social behavior [323]. Both are released from neurosecretory cells of the hypothalamus into the bloodstream via the capillary beds of the posterior pituitary gland. The most widely recognized functions of oxytocin are its effects on reproduction and social bonding, but it can also act as an anti-inflammatory, affecting the immune system [324]. Vasopressin is vital to regulating blood volume and salt concentrations in the body, by acting as an anti-diuretic [325].

While limited research has been conducted on the influence of selenium on oxytocin and vasopressin synthesis and signaling, there is some evidence of a relationship. In a recent case study, selenium deficiency was reported to have caused weak uterine contractions in a parturient mare [326]. Uterine contractions were typically stimulated by oxytocin [327]; however, in this case, oxytocin treatment failed to illicit a uterine response in the selenium deficient mare [326]. This might suggest that selenium is essential to oxytocin signaling downstream of oxytocin binding its receptor or, perhaps, to the expression or functionality of the oxytocin receptor itself. These possibilities are purely hypothetical, however, and the effects of selenium on oxytocin remain largely uncharacterized.

Long-term treatment of synthetic vasopressin has been associated with a significant decrease in renal clearance of selenium in ewes [328]. Following one week of vasopressin treatment, ewes showed a decrease in urinary output and no change to glomerular filtration rate or selenium concentration in urine, thereby indicating selenium retention. The authors of this report suggested that this reduction in renal clearance might be due to an increase in selenium solvent drag, caused by vasopressin treatment. They concluded that vasopressin (via renal function) might play a role in selenium maintenance in sheep. A study on the renal function of selenium-deficient mice reported no changes in the expression of vasopressin receptor in the kidneys, but no other aspects of vasopressin physiology were probed [329].

## 5. Signals from Body to Brain

The third aspect of homeostatic communication discussed in this review is the generation of signals in peripheral tissues that transmit information to the brain by acting on the hypothalamus. Much of the current knowledge of the role of selenium involves the production and secretion of hormones. Many past studies with selenium have focused on the intracellular signaling and physiological output in tissues targeted by these hormones, including the hypothalamus, and evidence of an effect on their production and release is limited. Still, there is potential for redox balance to impact the secretion of these hormones, leading to insufficient communication about homeostatic conditions to the hypothalamus [330,331,332,333]. This review mainly focuses on the hormones and nutrients that are relevant to energy homeostasis.

Insulin is the most heavily investigated homeostatic signal produced in the periphery in relation to selenium biology. Produced by β-cells in the pancreas, insulin is secreted during food consumption and provides an anorexigenic signal to the hypothalamus to suppress feeding and promote lipogenesis and the uptake of glucose by muscle. There are strong associations between selenium status, selenoprotein action, and type 2 diabetes (T2D), which have been thoroughly described in several recent review articles [24,43,190,334,335]. The nature of these interactions is complicated, as both low and high levels of selenium have been correlated with heightened T2D risk, and some results have shown a strong sexual dimorphism. Most of what is known about the relationship between selenium and insulin pertains to the development of systemic insulin resistance.

There is some evidence, however, that selenium can also affect insulin secretion through its influence on pancreatic physiology. For example, the synthesis and secretion of insulin by pancreatic β-cells requires constant disulfide bond formation and is, therefore, vulnerable to fluctuations in redox status [336]. Pancreatic β-cells also have high levels of SELENOP expression, which decreases under high glucose conditions and is upregulated by the β-cell toxin streptozotocin (STZ) [337]. It was proposed by one research group that SELENOP, acting as a hepatokine, could promote vulnerability to insulin resistance through a mechanism involving reductive stress [31,43,338]. In a study by Mita el al., excess SELENOP administration in mice decreased insulin levels in the pancreas and reduced the insulin response to glucose challenge, while causing a structural disarrangement of cells within pancreatic islets [339]. One potential underlying mechanism proposed by the authors for the abnormal islet morphology observed is a trans-differentiation of β-cells to α-cells. Conversely, treatment with a SELENOP-neutralizing antibody improved the metabolic phenotype of T2D model mice by upregulating insulin secretion. In a separate study from the same group, however, SELENOP was found to be essential to the health of MIN6 murine pancreatic cells through the prevention of ferroptotic cell death [164].

A study from Ueno et al. found that, although treatment with SeMet improved systemic glucose tolerance and alleviated oxidative stress in pancreatic islets of STZ-injected diabetes model mice, there was no observed effect on insulin storage and secretion [340]. It is important to note that the mice in this study were fed a selenium deficient diet and SeMet was orally administered only after the injection of STZ and, therefore, may not have had enough time to be properly metabolized and utilized by the pancreas. Nevertheless, the collective findings described here provide a strong argument for a direct effect of selenium on the ability of the pancreas to effectively use insulin to communicate with the brain.

The WAT-derived hormone leptin is a major regulator of energy balance that acts on neurons in the hypothalamus to suppress feeding behavior [5]. It was also discovered to be a thermokine that regulates sympathetic innervation of BAT thermogenesis and WAT lipolysis, through a hypothalamic circuit involving AGRP and POMC neurons [140]. The development of central leptin resistance is a hallmark symptom and agitator of obesity [341]. The most direct evidence of an ability of selenium to regulate leptin production involves PPAR-γ. Chemical agonism of PPAR-γ reduced leptin promoter activity and leptin production in mature cultured adipocytes and in in vivo diet-induced obese rats by inhibiting the CEBPα transcription factor [342,343,344,345]. Multiple groups reported that selenium supplementation upregulated PPAR-γ, while others put forth contrasting evidence that selenium repressed PPAR-γ [346,347,348]. These studies on the interactions between selenium and PPAR-γ utilized different tissue types, however, and none involved adipocytes. The only study done on adipocytes reported that sodium selenate application suppressed C3H10T1/2 cell differentiation into adipocytes by inhibiting *Ppar-γ* and leptin gene expression [349]. These results were recapitulated in 3T3-L1 preadipocytes, including the decrease in leptin gene expression caused by selenate, and an upregulation of SELENOS leading to reduced ER stress was pinpointed as the underlying mechanism [350].

There are several possible connections through which selenium could regulate leptin production through its influence on adipose tissue physiology [351]. Insulin stimulates leptin synthesis by promoting the eukaryotic initiation factor 4E (eIF-4E)-mediated translation of leptin mRNA [352]. Selenium could support this pathway by protecting against oxidative stress or through its insulin-like capabilities [353,354,355]. Another interesting connection lies in the finding that the statin medication atorvastatin inhibits leptin expression in cultured human smooth muscle cells through a mechanism involving ROS generation [356]. Moreover, the process of leptin synthesis itself was shown to produce ROS in rat portal vein organ cultures [357]. These correlations between altered redox states and changes in the regulation of leptin synthesis remain to be tested for an interaction with selenium and selenoproteins.

Beyond the research conducted on insulin and leptin, there are some reports demonstrating an influence of selenium on other homeostatic hormones. The adipose tissue hormone adiponectin, unlike leptin, correlates inversely with obesity and its secretion is stimulated by fat loss [358,359]. Adiponectin acts to improve insulin sensitivity by upregulating glucose and lipid metabolism and its function is, therefore, tied to T2D and other diseases affected by insulin sensitivity. When WAT deposits shrink, adiponectin inhibits lipolysis and constitutive KO of adiponectin allows for excessive lipolysis [360]. By binding its receptor in the hypothalamus, adiponectin is able to promote feeding behavior, as well as suppress GnRH secretion [361]. Studies in humans revealed that selenium supplementation (200 µg daily for 6 weeks) downregulated adiponectin receptor 1 (*Adipor1*) gene expression [362]. This change in mRNA levels was measured in white blood cells, however, and it remains to be seen whether that same regulation occurs within white adipocytes, which would indicate a potential pro-lipolytic mechanism. Interestingly, the insertion of a selenium atom into synthetic adenosine mimetics increased adiponectin secretion during lipogenesis in human mesenchymal stem cells, by improving the binding affinity for PPAR-γ [363]. Although this does not demonstrate an effect of naturally processed dietary selenium or selenoprotein action per se, the previously demonstrated ability of selenium to activate PPAR-γ in other tissues suggests that a similar mechanism in WAT cells could affect adiponectin secretion.

Animal studies provide further insight on the interactions between selenium and adiponectin. Two weeks of supplementation with sodium selenite in Otsuka Long-Evans Tokushima Fatty (OLETF) rats with spontaneous obesity decreased adipocyte size and adiponectin production, providing further evidence that selenium may directly inhibit adipocyte hypertrophy [364]. Supplementing STZ-induced diabetic mice with SeMet reduced adiponectin levels in adipocytes to improve glucose homeostasis, without affecting pancreatic insulin storage or secretion [340]. Yet another recent study on HFD-induced obese rats noted that sodium selenate delivered via drinking water upregulated adiponectin gene expression in epididymal fat [365]. Experiments with isolated adipocytes should be useful in determining whether these effects of selenium on adiponectin are the result of direct regulatory mechanisms within adipose tissue or are merely by-products of changes in whole-body energy homeostasis.

The orexigenic hormone ghrelin is produced by the gut during the “hunger” state to promote feeding [366]. Interestingly, ghrelin appears to have antioxidant and anti-inflammatory effects in various tissues, including in neurons [367,368]. To date, there is no report of a direct interaction with selenium and either ghrelin production or secretion. The addition of the antioxidant compounds resveratrol and curcumin to mouse gastric mucosal cell primary cultures, however, stimulated ghrelin secretion and blocked the inhibitory effect of glucose on ghrelin secretion [369]. Additionally, the inclusion of different NRF2 activators increased ghrelin secretion. Might the antioxidant activity of selenoproteins support ghrelin secretion through NRF2-mediated mechanisms?

**Figure 3:**
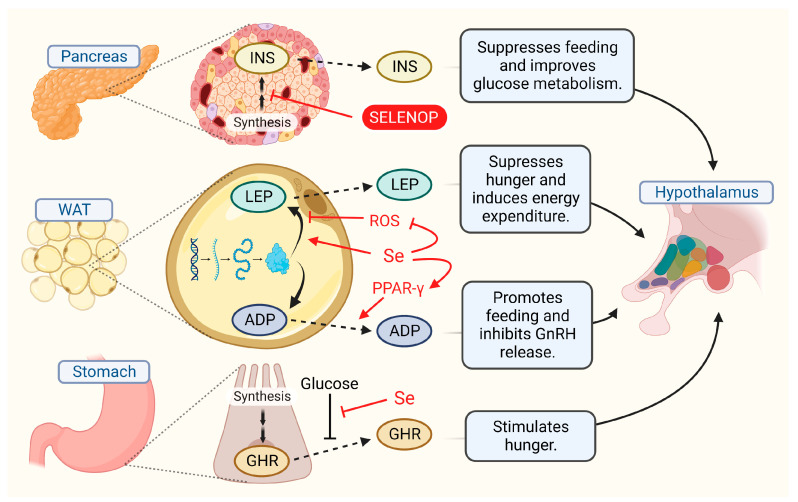
The potential effects of selenium on peripheral production of homeostatic signals to the hypothalamus. Although some studies report an insulin-like capability of selenium, the most direct evidence of an influence on pancreatic insulin (INS) secretion is the reported antagonism of insulin synthesis by the hepatokine selenoprotein P (SELENOP). In contrast, selenium plays a supportive role in the production and secretion of the hunger-suppressing adipokine leptin (LEP). Adiponectin, another adipose tissue-derived hormone, seems to be supported by selenium through a mechanism involving peroxisome proliferator-activated receptor-γ (PPAR-γ). Finally, selenium also seems to support ghrelin (GHR) secretion from the gut by countering the suppressive effect of excess glucose. ROS: reactive oxygen species, WAT: white adipose tissue. Effects of selenium are shown in red.

There are many correlations between selenium status and the release of homeostatic signals from peripheral tissues seen in animal and clinical studies. Further mechanistic studies are necessary, however, to determine whether these correlations are the result of direct interactions or are adaptations to overall changes in metabolic composition. Direct effects seem likely, as there are already well-characterized influences of selenium on the physiology of adipose tissue, the pancreas, and the gut [338,351,370]. Moreover, the synthesis and secretion of these hormones appear to be susceptible to redox-mediated changes. This specific intersection between selenium and energy homeostasis is surprisingly under-investigated, but future work will shed light on the contributions of selenium to the ability of the body to generate homeostatic signals to the brain. The current knowledge on this subject is depicted in Figure 3.

## 6. Concluding Remarks

The role of selenium in energy homeostasis is complex and the literature is full of somewhat contrasting data [189,371,372]. Conflicting reports could be the result of many variables, such as selenium status and confounding health conditions in the case of clinical studies, where patients were supplemented with selenium, as well as the specific form of selenium and the route of delivery used in animal studies. Based on the insights provided in this review, the mechanisms through which selenium can influence homeostatic communication are diverse, tissue-specific, and, sometimes, counter-effective. For example, disrupting the intracellular selenium recycling process broadly in the mouse hypothalamus is obesogenic, but the result of an AGRP neuron-specific disruption is a lean phenotype [27,34,142]. Thus, tissue-specific deficiency and mis-utilization of selenium can underlie a whole-body state of metabolic disorder. Such conditions cannot be ruled out simply because markers of selenium status and intake are thought to indicate ‘sufficiency’.

Selenium is metabolized, distributed, and utilized throughout the body by a unique system of enzymes, factors, and receptors. Local impairments of the molecular machinery involved could result in tissue-specific deficiencies. Indeed, selenium dyshomeostasis, as opposed to an overall deficiency or over-consumption, has been proposed as a major contributor to human disease pathology, particularly neurodegeneration [373,374,375]. The dyshomeostasis hypothesis helps reconcile previous conflicting reports on the correlations between selenium status and disease severity, as it takes into consideration tissue-specific effects. Fully elucidating the interactions between selenium and homeostatic communication outlined in this review is necessary for a better understanding of the multifactorial nature of metabolic disease. Such a course of investigation seems ever more relevant as the field increasingly focuses on precision nutrition [376,377]. If the transport of selenium across the blood–brain barrier becomes impaired in the hypothalamus, resulting in a local deficiency and greater susceptibility to leptin resistance, will selenium supplementation help counteract the problem? Will it instead promote SELENOP-mediated insulin resistance [378]? Questions such as these implicate the need for targeted approaches in the development of selenium-based therapy. Addressing these perplexities should remain a top priority for researchers in the field.

## Figures and Tables

**Table 1:** Selenoprotein names and functions.

Selenoprotein	Abbreviation(s)	Function/Reactions Catalyzed
lodothyronine deiodinases (1–3)	DIO (1–3)	Types 1 and 2 activate thyroid hormones (T4 to T3) and type 3 deactivates (T3 to T2, or T4 to rT3). Type 2 localizes to the ER.
Glutathione peroxidases (1–4, 6)	GPX (1–4, 6)	Reduces hydrogen peroxide species. Type 1 is present in cytosol. Type 4 reduces phospholipid hydroperoxides.
Methionine sulfoxide reductase B1	MSRB1	Reduces sulfoxidated methionipes.
Selenophosphatase synthetase 2	SEPHS2	Synthesis of selenophosphate to support selenoprotein synthesis.
Selenoprotein F	SELENOF	Thiol-disulfide oxidoreductase in the ER.
Selenoprotein H	SELENOH	Localized to the nucleus. Thought to conduct redox sensing to support transcription.
Selenoprotein I	SELENOI	Ethanolamine phosphotransferase to support the synthesis of phosphatidylethanolamine.
Selenoprotein K	SELENOK	Palmitoylation of inositol triphosphate receptors to facilitate store-operated Ca^2+^ entry from the ER.
Selenoprotein M	SELENOM	Thio-disulfide oxidoreductase in the ER.
Selenoprotein N	SELENON	Oxidoreductase that senses ER luminal Ca^2+^ levels.
Selenoprotein O	SELENOO	Localized to the mitochondrion.
Selenoprotein P	SELENOP	Secretory glycoprotein that delivers selenium to cells throughout the body.
Selenoprotein S	SELENOS	Participates in ER associated protein degradation.
Selenoprotein T	SELENOT	Thought to regulate Ca^2+^ homeostasis in the ER.
Selenoprotein V	SELENOV	Regulates O-GlcNAcylation.
Selenoprotein W	SELENOW	Proposed to have antioxidant function.
Thioredoxin reductases (1–3)	TXNRD (1–3)	Reduction of oxidized thioredoxin. Type 1 localizes to cytoplasm, type 2 to mitochondria.

## Data Availability

Not applicable.
